# Supplementary material for: Al[18F]F-NOTA-Octreotide Is Comparable to [68Ga]Ga-DOTA-TATE for PET/CT Imaging of Neuroendocrine Tumours in the Latin-American Population
Source: Cancers (Basel). 2023 Jan 10;15(2):439. doi: 10.3390/cancers15020439 (PMC9856643; doi:10.3390/cancers15020439)
Supplement: Supplementary file 1 [file cancers-15-00439-s001.zip › cancers-2109817-supplementary.pdf]

cancers

MDPI

Article

Al[18F]F-NOTA-Octreotide is noninferior to [68Ga]Ga-DOTA-TATE for PET/CT imaging of advanced neuroendocrine tumours in the Latin-American population

Arlette Haeger[1,5], Cristian Soza-Ried[1,2,5], Vasko Kramer[1,2], Ana Hurtado de Mendoza[1], Elisabeth Eppard[1,3], Noémie Emmanuel[4], Johanna Wettlin[1], Horacio Amaral[1,2], and René Fernández[1]

1) Nuclear Medicine and PET/CT center PositronMed, 7501068 Providencia, Santiago, Chile
2) Positronpharma SA, 7500921 Providencia, Santiago, Chile
3) Department of Nuclear Medicine, University Hospital Magdeburg, Germany
4) Ion Beam Applications, Louvain-la-Neuve, Belgium
5) A H. and C.S.R. contributed equally to this work.
* Correspondence: ahaeger@positronmed.cl

Extended materials and methods - Radiochemistry:

Radiolabeling of Al[18F]F-NOTA-Octreotide was performed in similar conditions described by McBride et al. using an automated procedure validated on a cassette-based radiosynthesizer (Synthera®+, IBA Radiopharma Solutions, Louvain-la-Neuve, Belgium). A standard IFP for nucleophilic labelling was customized according to figure S1 to fit the needs of the process. 100±51 GBq ^{18}F-fluoride (IBA cyclone 18/18) in 2.3 mL water were trapped on a strong anion exchange cartridge (QMA Light, Waters, conditioned with 5 mL 0.9% sodium chloride, 10 mL ultrapure water, 3 x 30 mL air), rinsed with 4.0 mL ultrapure water from vial 5 and eluted with 0.5 mL Eluent (300 µL 0.9% sodium chloride and 200 µL ethanol) from Vial 1 into the reactor. A solution 200 µL NOTA-Octreotide (2.0 mg/mL in ultrapure water), 33 µL $AlCl_3$ stock solution (0,01 M in 100 mM sodium acetate buffer pH 4.1) and 650 µL acetonitrile (containing 6.6 mg ascorbic acid in 16.2 µL water) were added to the reactor from vial 3. The reaction was heated to 100 °C for 8 min, cooled to room temperature, diluted with 7-8 mL ascorbate-buffer (28.8 mg/mL in water) from vial 4 and passed through a C18 cartridge. The C18 cartridge was rinsed twice with additional 6 mL of ascorbate-buffer and eluted with 3.0 mL 66% ethanol from vial 2 and into 19 mL ascorbate-buffer. The final product was passed through a sterile filter (Millex-GS, 0.22 µm) for final formulation.

Table S1: Representative batches (last 5 executed) for the production of Al[18F]F-NOTA-Octreotide

Batch N°	1	2	3	4	5	Mean	±	SD
Batch Number	FAN220813_0801	FAN220907_P2	FAN220929_0973	FAN221012_1024	FAN221020_1054	ND		
Date	13.08.2022	07.09.2022	29.09.2022	12.10.22	20.10.2022	ND		
Operator	LC					ND		
Module	S+					ND		
Method	AlF-NOTA-Octreotide v1.0					ND		
Start of Synthesis (Elution)	11:38	18:58	9:28	11:37	9:50	ND		
End of Synthesis	12:06	19:27	10:01	12:11	10:22	ND		
Duration / min	28	29	33	34	32	31.2	±	2.6
Start activity / GBq	40.8	61.7	100.6	169.5	128.1	100	±	51
Product activity / GBq	8.1	23.1	44.1	54.6	45.0	35	±	19
Activity yield / %	19.8%	37.5%	43.9%	32.2%	35.1%	33.7%	±	8.9%
Product volume / mL	22	22	22	22	22	22	±	0
Activity concentration / GBq/mL	0.4	1.1	2.0	2.5	2.0	1.6	±	0.9
NOTA-Octreotide concentration / ug/mL	18.2	18.2	18.2	18.2	18.2	18.2	±	0.0
Specific activity @EOS / MBq/µg	20.2	57.8	110.3	136.5	112.4	87.4	±	47.3

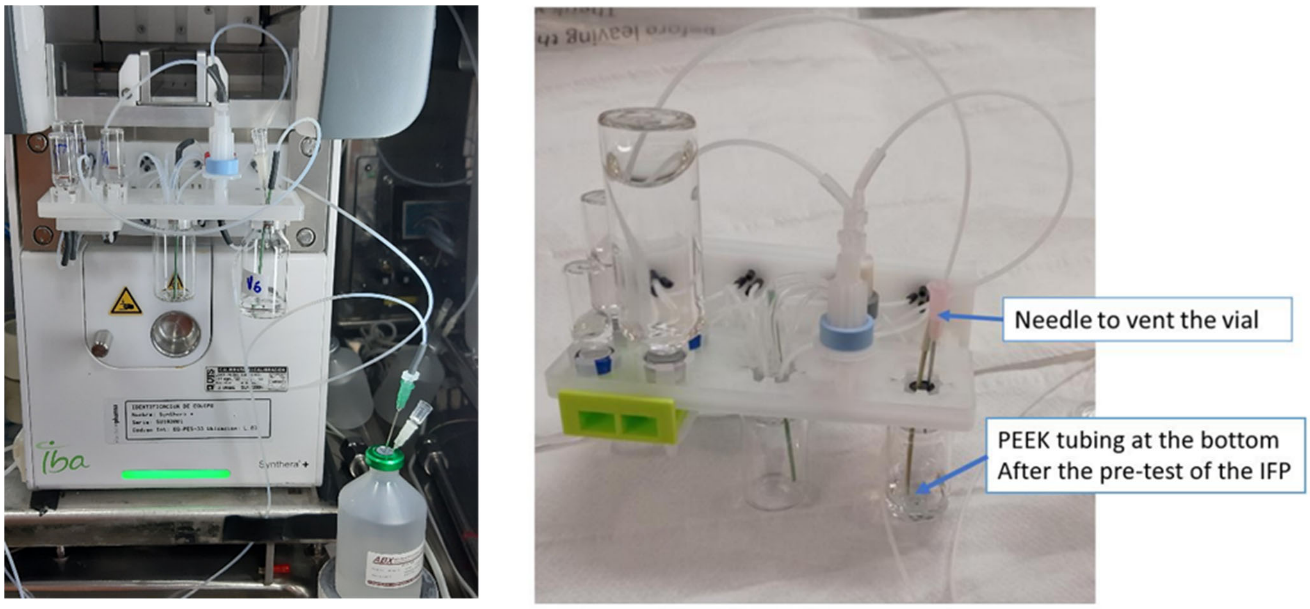

Figure S1: Synthera®+ Synthesizer with loaded IFP and reagents (left), IFP with reagents and cartridges

Radiochemical purity testing was performed on an analytical HPLC system (Agilent 1260 series; Gabi Star radiodetector and Gina Star v6.0 evaluation software, Elysia-Raytest, Angleur, Belgium). Column: Phenomenex Luna C18 150x4.6mm, solvent A: 0.15 M ammonium acetate buffer pH 5.5, solvent B: acetonitrile, column flow: 1.5 mL/min, UV-wavelength: 220 nm, injection volume: 20 µL, gradient: 20% B (0-2 min), 20-30% B (2-12 min), 30% B (12-15 min), 30-20% B (15-15.5 min), 20% B (15.5-18 min).

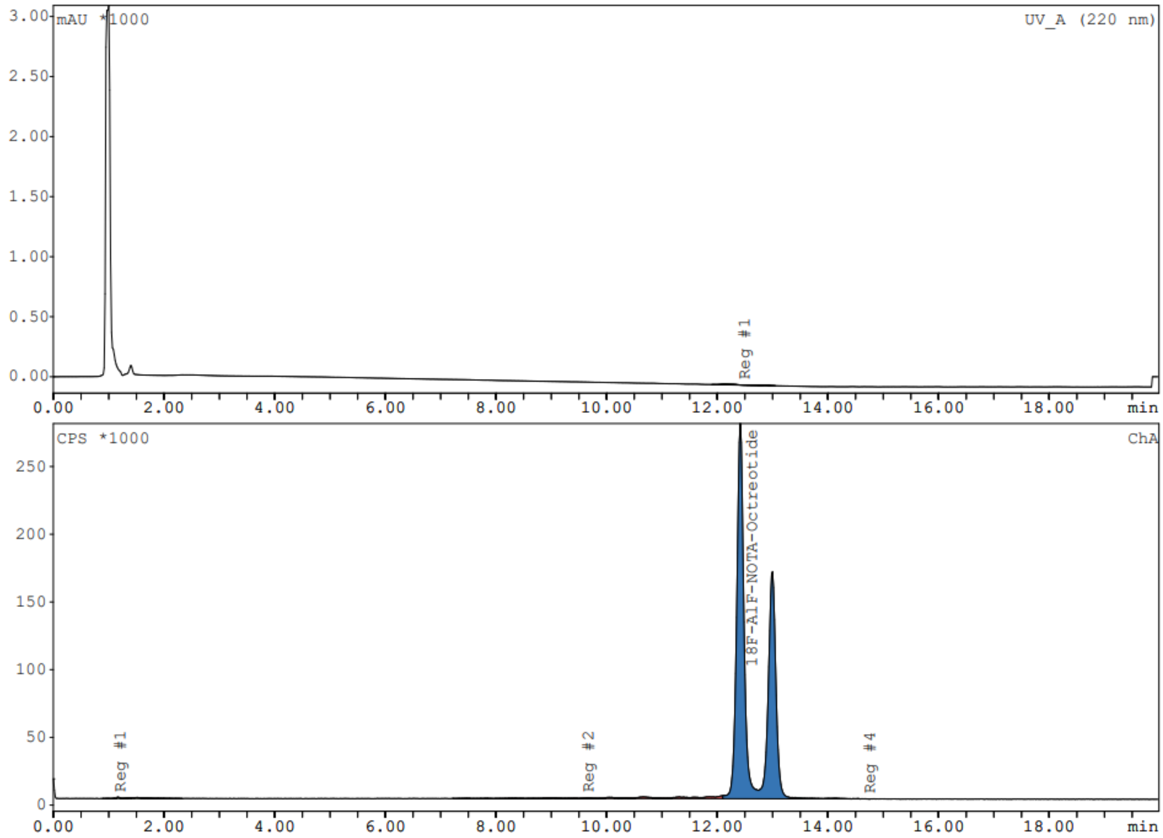

Figure S2: Representative HPLC-Chromatogram for Al[^{18}F]F-NOTA-Octreotide; UV signal (upper row) and radioactivity (lower row). Product elutes at 12.4 min (isomer 1) and 13.0 min (isomer 2) and main impurity is [^{18}F]F$^-$ eluting at 1.2 min. The UV peak at t=0,8 corresponds to ascorbate.

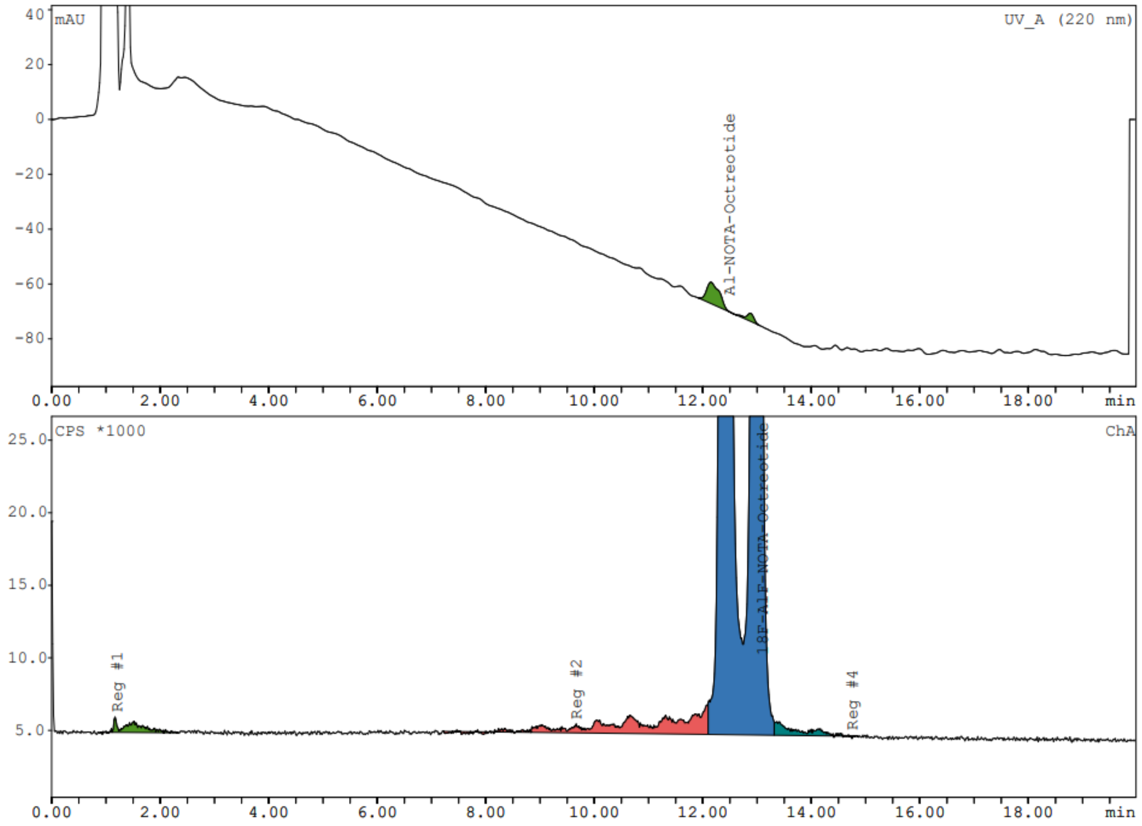

Figure S3: Zoom of HPLC-Chromatogram in figure S2. for Al[^{18}F]F-NOTA-Octreotide; UV signal (upper row) and radioactivity (lower row). Product elutes at 12.4 min (isomer 1) and 13.0 min (isomer 2) and main impurity is [^{18}F]F$^-$ eluting at 1.2 min. The UV peak at t=0,8 corresponds to ascorbate.

Extended materials and methods – Inclusion/Exclusion Criteria:

Inclusion Criteria:

- Age between 18 and 75 years.
- Signed informed consent.
- Subject is diagnosed with a well-differentiated neuroendocrine tumour (Ki-67 <20), new or in follow-up, of unknown primary origin, gastroenteropancreatic, pulmonary or neural.
- The subject is judged to be in good general condition by the investigator based on clinical history, physical examination including vital signs and clinical laboratory tests, in addition to the diagnosis of neuroendocrine tumour.
- Subject must have at least 5 positive lesions on routine PET/CT study with [^{68}Ga]Ga-DOTA-TATE performed within two weeks prior to the inclusion visit. A positive lesion is defined as marker uptake above background because of the presence of tumour neuroendocrine cells, evaluated by a nuclear medicine physician.
- Women at risk for pregnancy must have a recent (not > 3 days) blood human chorionic gonadotropin (hCG) test with values <5mIU/mL that exclude pregnancy.

Exclusion Criteria:

- Subject has a previous or current recurrent or chronic disease, other than a neuroendocrine tumour, with high risk of interfering with trial evaluation at the investigator's discretion, e.g., known gastrointestinal, hepatic, renal, cardiovascular, metabolic, or hormonal disease, cancer, severe neurological or seizure disorder, or any psychiatric disease.
- Subject has been exposed to ionizing radiation (> 1 mSv) in other research studies within the past 12 months.
- Subject suffers from claustrophobia or cannot tolerate confinement during PET/CT scanning procedures; subject cannot remain still for 60 minutes inside the scanner.
- Subject does not understand the study procedure.

- Subject is unwilling or unable to perform all study procedures or is otherwise deemed unsuitable by the principal investigator.
- Women at risk of pregnancy or desire to become pregnant before 3 months after the study.
- Subject has recently participated (< 30 days) or is concurrently participating in another prospective interventional clinical trial.
- Subject underwent surgery between screening and the inclusion visit.
- Subject is mentally or legally incapacitated.

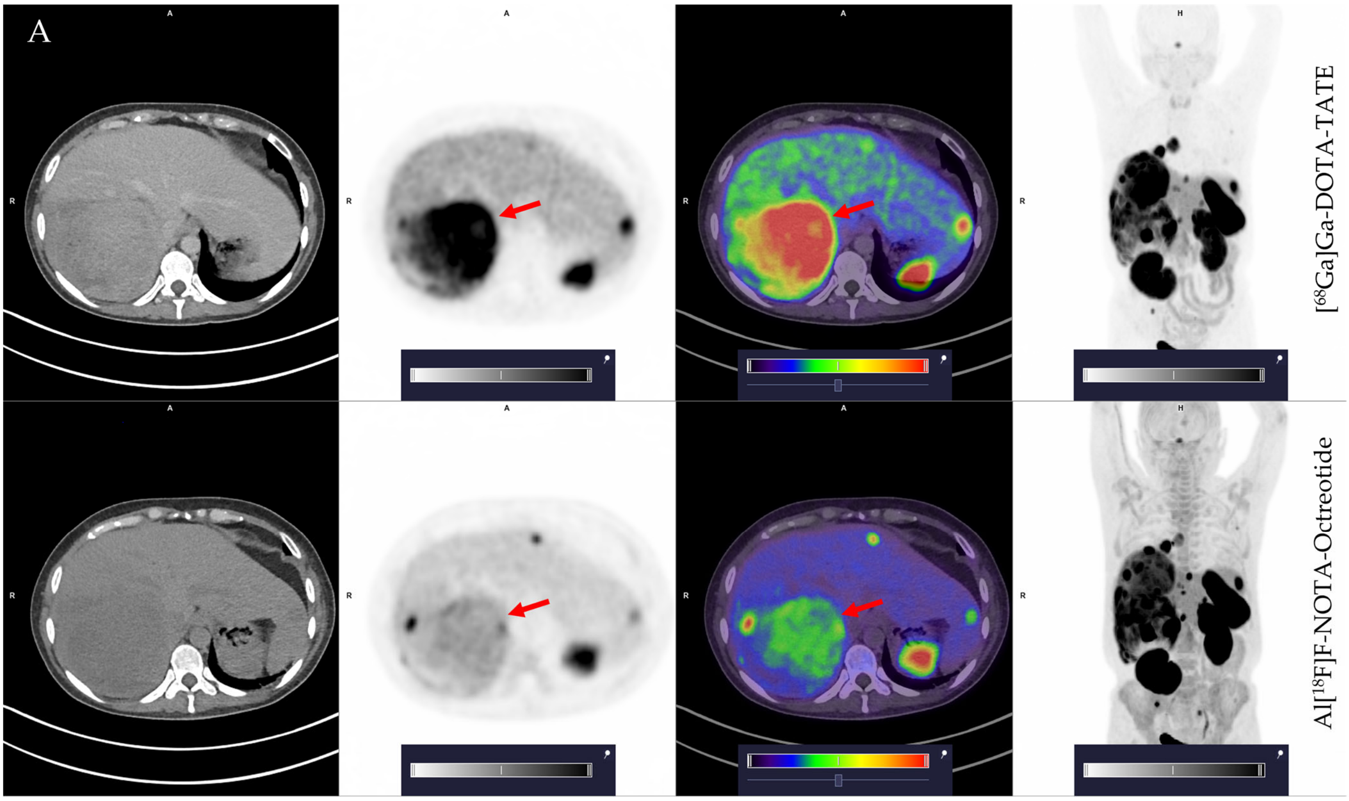

Figure S4: A) Patient ID 2, (45 y, female) with a dominant right liver lesion (red arrow) with significantly higher uptake with [^{68}Ga]Ga-DOTA-TATE (upper row) versus Al[^{18}F]F-NOTA-Octreotide (lower row).

Table S2: Release specifications and overall QC results for Al[^{18}F]F-NOTA-octreotide - There is no US Pharmacopoeia monograph available. Where applicable, the specifications have been based on the existing USP for [^{68}Ga]Ga-DOTA-TATE.

Parameter	Method	Acceptance Criteria	Overall results (Mean±SD)
**Appearance**	Visual	Clear, colorless, or slightly yellow solution	Clear, colorless solution
**Radionuclide Identity (keV)**	Gamma-ray spectrometry	Only peaks at 511 and 1022 keV gamma-energy	522±7
**Radionuclide Identity (min)**	Approximate half-life	105-115 min	106.8±0.7
**pH**	pH indicator strips	4.5-8.5	5.0±0.4
**Ethanol (ppm)**	GC	< 79.000 ppm (10 %)	40278±16377
**Acetonitrile (ppm)**	GC	< 410 ppm	24±18
**Radiochemical purity (%)**	HPLC	< 5 % [^{18}F]F$^-$ + Al[^{18}F]F^{2+}	1.9±2.3
**Radiochemical purity (%)**	HPLC	> 91 % Al[^{18}F]F-NOTA-Octreotide	97.2±3.8
**Bacterial endotoxins (EU/mL)**	LAL test	< 175/V; 17.5 EU/mL	<1.0±0.0
**Sterility**	Direct inocculation (post release)	Sterile	No growth
**Radionuclidic purity (%)**	Gamma spectrometry (post release)	>99.9%	99.98±0.01
